# The Western Texas Medical Association

**Published:** 1900-11

**Authors:** 


### The Western Texas Medical Association.

The Western Texas Medical Association held its Twenty-fourth Annual Meeting, in San Antonio, October 25th (ult.), as per announcement, and carried out an interesting program; winding up with the usual banquet, toasts and speech-making. Dr. W. E. Luter, the long-time Secretary, now First Vice-President, sends us the following brief report:

Dr. A. S. McDaniel, of San Antonio, read a paper on the "Injection of Cocaine into the Spinal Canal to Render Labor, as well as Operations on the Extremities, Painless." Dr. McDaniel has just returned from a short trip to Paris, and while there witnessed several cervical operations on patients who had received the spinal cocaine injections. He reported that the method seemed to give satisfactory results, and he was favorably impressed with it. Nearly everyone present at the meeting took part in the discussion of the subject, and the general opinion was, that the injection of cocaine into such a sacred canal as is the spinal canal for the purpose of rendering labor and operations painless, is attented with too great a risk of infection and danger to the patient, and it was their belief that the method would never become popular.

Dr. S. Burg, the President, read an exceedingly interesting and practical paper on "Anesthesia in Labor."*

After Dr. Burg's paper the minutes of the last annual meeting were read, and adopted, and the applications for membership of Drs. R. M. Scott and G. L. Moody were presented and referred to the Board of Censors.

Election of officers resulted in the following:

President.—Dr. A. Garwood, New Braunfels, Texas.

First Vice-President.—Dr. Wm. E. Luter, San Antonio, Texas.

Second Vice-President.—Dr. R. L. Withers, San Antonio, Texas.

Secretary and Treasurer.—Dr. R. Dinwiddie, San Antonio, Texas.

Board of Censors.—S. Burg, San Antonio, Texas, Chairman; E. Hertzburg, San Antonio, Texas; C. E. R. King, San Antonio, Texas; B. F. Kingsley, San Antonio, Texas; A. S. McDaniel, San Antonio, Texas.

